# PD-L1 in the palm of your hand: palmitoylation as a target for immuno-oncology

**DOI:** 10.1038/s41392-019-0053-x

**Published:** 2019-06-07

**Authors:** Andreas von Knethen, Bernhard Brüne

**Affiliations:** Institute of Biochemistry I - Pathobiochemistry, Faculty of Medicine, Goethe-University Frankfurt, Theodor-Stern-Kai 7, 60590 Frankfurt am Main, Germany

**Keywords:** Translational research, Molecular medicine

**Fig. 1:**
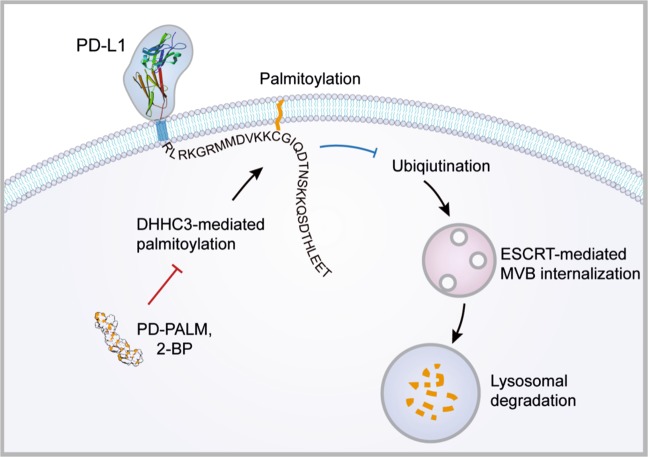
**Palmitoylation regulates PD-L1 stability and trafficking**. This lipid modification can be blocked by PD-PALM or 2-BP to enhance anticancer immunity

A recent study by Yao et al. published in *Nature Biomedical Engineering* showed the importance of palmitoylation in regulating PD-L1 protein stability and trafficking.^1^ These researchers developed a competitive inhibitor that blocks PD-L1 palmitoylation, which may provide a new strategy for targeting PD-L1 or other palmitoylated membrane proteins (Fig. 1).

Immune checkpoint blockade (ICB) has been intensively studied as an approach for cancer therapy, with over 1300 records for PD-1 and PD-L1 on the ClinicalTrials.org website. Despite clinical trial successes, ICB therapy still has major challenges to overcome, such as the relatively low response rate, acquired resistance, and occasional fatal adverse effects.^2^

PD-L1 expression, as a biomarker for ICB therapy, varies considerably among tumor types, stages, cases and samples and may also change during therapy. Moreover, our understanding of the molecular regulation of PD-1/PD-L1 is still limited.^3,4^

A recent study by Jie Xu’s group at Shanghai Jiao Tong University (Renji Hospital, State Key Laboratory for Oncogenes and Related Genes) and Hubing Shi’s laboratory at Sichuan University (West China Hospital, State Key Laboratory for Biotherapy, China) revealed a new mechanism governing the stability of PD-L1.^1^ The researchers found that palmitoylation decreases the lysosomal degradation of PD-L1, which is a crucial target for ICB therapy, and developed a targeting peptide termed PD-PALM, which seems to be a first-in-class molecule that competitively inhibits PD-L1 palmitoylation.

Palmitoylation is a reversible lipid modification on proteins that controls a wide range of protein functions, including trafficking, activity, stability, and membrane association.^5^ Protein palmitoylation is catalyzed by DHHC (Asp-His-His-Cys) enzymes, whereas depalmitoylation is mediated by acyl-protein thioesterase (APT). At least 25 DHHC enzymes have been characterized in the human genome, with different substrate specificities and subcellular localization patterns. Palmitoylation has been found to regulate multiple cancer-related proteins such as EGFR, Ras, Wnt, etc. It remains largely unknown whether palmitoylation controls immune checkpoint signaling.

The researchers found that DHHC3-dependent palmitoylation of PD-L1 inhibited its ubiquitination, which is required for the ESCRT-mediated internalization of PD-L1 into multivesicular bodies (MVB) and lysosomes. Using 2-BP, a small-molecule inhibitor of palmitoylation, the authors demonstrated that blocking palmitoylation efficiently induced the lysosomal degradation of PD-L1 in tumor cells. This treatment enhanced the tumor-specific cytotoxicity of T-cells both in vitro and in vivo. Their experiments suggest a new mode of action of palmitoylation inhibitors, as the injection of 2-BP, but not anti-PD-L1 antibody, efficiently decreased the expression of PD-L1 in MC38 tumor tissues.

One major challenge in targeting palmitoylation is the lack of specificity; existing palmitoylation inhibitors are known to target all DHHC members. In addition to its PD-L1-related effects, 2-BP may also cause unwanted effects due to its potential suppression of other palmitoylated proteins. To improve targeting specificity, the authors developed a competitive inhibitor of PD-L1 termed PD-PALM. This development was inspired by the finding that DHHC substrate specificity is determined by the peptide sequence surrounding the palmitoylated cysteine residue. They designed a chimeric peptide comprising a cell-penetrating peptide and a peptide fragment from PD-L1 encompassing the Cys272 residue. Treatment of cancer cells with PD-PALM significantly decreased the palmitoylation and expression of PD-L1 in tumor cells, supporting their concept of targeting palmitoylation with selective competitive inhibitors.

Their research shed light on a new modification of PD-L1, with relevance to PD-L1 expression and function. More importantly, they developed a competitive inhibitor that targets the palmitoylation of PD-L1. Their translational research suggests that PD-L1, potentially along with other membrane proteins, is druggable based on evidence obtained after interfering with palmitoylation, a dynamic lipid modification process. It will be very interesting to translate these findings into the clinic.
